# Comparative genome analysis of pathogenic and non-pathogenic *Clavibacter* strains reveals adaptations to their lifestyle

**DOI:** 10.1186/1471-2164-15-392

**Published:** 2014-05-22

**Authors:** Joanna Załuga, Pieter Stragier, Steve Baeyen, Annelies Haegeman, Johan Van Vaerenbergh, Martine Maes, Paul De Vos

**Affiliations:** Laboratory of Microbiology, Department of Biochemistry and Microbiology, Ghent University, K.L. Ledeganckstraat 35, Gent, B-9000 Belgium; Plant Sciences Unit - Crop Protection, Institute for Agricultural and Fisheries Research - ILVO, Burg. Van Gansberghelaan 96, Merelbeke, B-9820 Belgium; BCCM/LMG Bacteria collection - Laboratory of Microbiology Department of Biochemistry and Microbiology, Ghent University, K.L. Ledeganckstraat 35, Gent, B-9000 Belgium

**Keywords:** Non-pathogenic *Clavibacter*, Bacterial wilt and canker, Tomato seeds, Genome sequencing, Quarantine, Plant pathogen

## Abstract

**Background:**

The genus *Clavibacter* harbors economically important plant pathogens infecting agricultural crops such as potato and tomato. Although the vast majority of *Clavibacter* strains are pathogenic, there is an increasing number of non-pathogenic isolates reported. Non-pathogenic *Clavibacter* strains isolated from tomato seeds are particularly problematic because they affect the current detection and identification tests for *Clavibacter michiganensis* subsp. *michiganensis* (Cmm), which is regulated with a zero tolerance in tomato seed. Their misidentification as pathogenic Cmm hampers a clear judgment on the seed quality and health.

**Results:**

To get more insight in the genetic features linked to the lifestyle of these bacteria, a whole-genome sequence of the tomato seed-borne non-pathogenic *Clavibacter* LMG 26808 was determined. To gain a better understanding of the molecular determinants of pathogenicity, the genome sequence of LMG 26808 was compared with that of the pathogenic Cmm strain (NCPPB 382). The comparative analysis revealed that LMG 26808 does not contain plasmids pCM1 and pCM2 and also lacks the majority of important virulence factors described so far for pathogenic Cmm. This explains its apparent non-pathogenic nature in tomato plants. Moreover, the genome analysis of LMG 26808 detected sequences from a plasmid originating from a member of *Enterobacteriaceae*/*Klebsiella* relative. Genes received that way and coding for antibiotic resistance may provide a competitive advantage for survival of LMG 26808 in its ecological niche. Genetically, LMG 26808 was the most similar to the pathogenic Cmm NCPPB 382 but contained more mobile genetic elements. The genome of this non-pathogenic *Clavibacter* strain contained also a high number of transporters and regulatory genes.

**Conclusions:**

The genome sequence of the non-pathogenic *Clavibacter* strain LMG 26808 and the comparative analyses with other pathogenic *Clavibacter* strains provided a better understanding of the genetic bases of virulence and adaptation mechanisms present in the genus *Clavibacter.*

**Electronic supplementary material:**

The online version of this article (doi:10.1186/1471-2164-15-392) contains supplementary material, which is available to authorized users.

## Background

*Clavibacter* is generally considered a genus of plant pathogens, but ecological surveys suggest that environmental, non-pathogenic *Clavibacter* occur more commonly than previously thought [1, 2]. Generally these non-pathogenic isolates are overlooked since diagnostic procedures focus on pathogenic strains. Just recently, studies were undertaken to initiate the characterization of these non-pathogenic isolates [3, 4]. The main objective of this study was to investigate key genomic features of non-pathogenic *Clavibacter* isolated from tomato seeds. These strains tend to be misidentified as Cmm in serological and molecular tests used in seed assays. Their high genetic and phenotypic similarity to pathogenic Cmm strains hampers a clear judgment on seed health.

The majority of non-pathogenic *Clavibacter* strains isolated from tomato seeds exhibit similar cell and colony morphology to the genuine Cmm [5]. Because of the common biological origin (tomato seed), high sequence similarities and similar physiological characteristics, the non-pathogenic *Clavibacter* strains are suggested to be the most related to Cmm. Initial *in planta* experiments demonstrated that this group of isolates is not pathogenic to the tomato plant and they do not colonize the vascular tissues of tomato [4]. Non-pathogenic clavibacters neither induce a hypersensitive reaction (HR) after infiltration of *Nicotiana tabacum* and *Nicotiana benthamiana* leaves [3], nor when inoculated to *Mirabilis jalapa* (J. Van Vaerenbergh, personal communication). Furthermore, a majority of these strains is lacking one or both Cmm plasmids carrying important virulence factors.

So far there is very little information available on non-pathogenic *Clavibacter* strains isolated from tomato seeds. Reports concerning the ecological niche, survival abilities or nutritional requirements are lacking. Knowledge about the biology of these strains is limited, not only because they were only recently identified as constituting a separate *Clavibacter* group but also because their significance in the Cmm identification procedure has not been evaluated previously. Their ecological niches remain unknown; routes of transmission and possible sources of these strains have not yet been recognized.

High genetic and phenotypic similarities of non-pathogenic *Clavibacter* and pathogenic Cmm strains are the main reasons for their misidentifications as Cmm in the currently recommended detection/identification tests for Cmm in tomato seeds [6]. Cross-reactions with antisera specific for Cmm and/or positive PCR reactions with primers used for identification of Cmm illustrate the proximity of surface antigens and genomic sequences of non-pathogenic seed-borne *Clavibacter* to the pathogenic Cmm [3]. Recent studies demonstrated that neither PCR assays based on commonly used 16S rRNA genes or ITS region, nor those designed for the detection of known virulence factors are specific to only pathogenic Cmm [3, 7]. Furthermore, some non-pathogenic *Clavibacter* strains showed fainter PCR amplicons on the gel impeding the correct interpretation of the results [4]. Taxonomically, these non-pathogenic clavibacters from tomato seeds are distinct from all *Clavibacter* subspecies (based on the analysis of housekeeping genes *gyrB* and *dnaA*) [4].

Recent developments in the field of molecular biology and sequencing allowed generating complete genome sequences and subsequently determining metabolic traits for many organisms. Complete genome sequences of Cmm NCPPB 382 [8], Cms ATCC 33113 [9] and Cmn NCPPB 2581 (released without publication) provide genetic information that allows for comparative studies and helps to better understand their pathogenicity characteristics and host adaptation. However, no information is available about the genome content of non-pathogenic *Clavibacter* strains, which could deliver some informative insights into the differences in virulence determinants, genetic content and adaptation to a lifestyle in their natural ecological niche(s). Genome comparison between pathogenic and non-pathogenic strains belonging to the same species is an important and valuable approach to identify genes that may contribute to virulence and general fitness of the organism.

In this report we present the genome analysis of non-pathogenic *Clavibacter* LMG 26808 isolated from tomato seed. The specific purposes of this study were a) to generate a draft genome sequence of this strain, b) to analyze it for virulence-related gene content by comparing it to the available genome of the pathogenic *Clavibacter michiganensis* subsp. *michiganensis* (Cmm) NCPPB 382, c) to perform a comparative analysis with the genomes of Cmm (NCPPB 382) [8], *Clavibacter michiganensis* subsp. *nebraskensis* (Cmn) (NCPPB 2581, released without publication) and *Clavibacter michiganensis* subsp. *sepedonicus* (Cms) (ATCC 33113) [9], pathogenic to tomato, maize and potato, respectively, d) to search for adaptations to a non-pathogenic lifestyle.

## Methods

### Strains and DNA extraction

Non-pathogenic *Clavibacter* sp. LMG 26808 was received as isolate PD 5684 from Naktuinbouw, The Netherlands. It was obtained in dilution plating on semi-selective media according to the current method for detection of Cmm in tomato seeds recommended by the International Seed Federation (ISF) [6]. LMG 26808 is phenotypically similar to Cmm on SCMF and CMM1T and was identified as Cmm in commonly practiced PCR tests but showed no pathogenicity in tomato plants [3, 4]. LMG 26808 was aerobically grown on MTNA (mannitol, trimethoprim, nalidixic acid, amphotericin) medium without antibiotics at 25°C for 24 h-48 h [10]. Stock cultures were stored at −80˚C in Microbank^TM^ beads (Pro-Lab Diagnostics, Canada). Total genomic DNA was extracted according to the guanidium-thiocyanate-EDTA-sarkosyl method described by Pitcher [11], which was adapted for Gram-positive bacteria by a pre-treatment with lysozyme (5 mg/μl lysozyme in TE buffer) and incubation for 40 minutes at 37°C.

### Plasmid extraction

Isolation of plasmid DNA was based on the alkaline method of Anderson and McKay [12]. Agarose gel electrophoresis was performed in a Tris acetate buffer containing 40 mM Tris, 20 mM acetic acid, and 2 mM Na_2_EDTA (pH 8.1). Gels contained 0.8% agarose and electrophoresis was performed at 55 V for 16 hrs at 4˚C. Gels were stained with ethidium bromide 0.5 μg/ml.

### Genome sequencing

Library preparation and genome sequencing was performed by BaseClear (Leiden, The Netherlands). High-molecular weight genomic DNA was used as input for library preparation using the Illumina TruSeq DNA library preparation kit (Illumina). Briefly, the gDNA was fragmented and subjected to end-repair, A-tailing, ligation of adaptors including sample-specific barcodes and size-selection to obtain a library with median insert-size around 300 bp. After PCR enrichment, the resultant library was checked on a Bioanalyzer (Agilent) and quantified. The libraries were multiplexed, clustered, and sequenced on an Illumina HiSeq 2000 with paired-end 50 cycles protocol. The sequencing run was analyzed with the Illumina CASAVA pipeline (v1.8.2). The raw sequencing data produced was processed removing the sequence reads which were of too low quality (only “passing filter” reads were selected) and discarding reads containing adaptor sequences or PhiX control with an in-house filtering protocol.

A paired-end (PE) DNA library with a mean insert size of 300 bp was sequenced with average reads of 101 bp on an Illumina Genome HiSeq2000 (Illumina Inc.). Next, a mate-paired (MP) DNA library with a mean insert size of 3800 bp was sequenced with average reads of 51 bp on an Illumina Hiseq2000 (Illumina Inc.). Automatic trimming (based on a threshold of *Q* = 20) and assembly was performed using CLC Genomics Workbench v5.0. An initial *de novo* assembly was performed in CLC Genomics Workbench v5.0 using the quality trimmed and paired reads from the PE and MP reads. All contigs shorter than 200 bp were discarded. Remaining N-nucleotides in the scaffolds, introduced during scaffolding, were removed from the final sequence by breaking up the scaffolds back into contigs where they were encountered. The quality of the final draft genome sequence was compared to the initial PE-based *de novo* assembly through comparative read-mapping in CLC Genomics Workbench v5.0 using the trimmed read sets. Contigs were ordered automatically with MAUVE [13] and manually with Artemis [14] by comparing with Cmm NCPPB 382.

### Genome annotation

Functional annotation and metabolic reconstruction were performed with (1) the Rapid Annotation Subsystem Technology (RAST) server [15], using Glimmer [16] for gene calling and allowing frameshift correction, backfilling of gaps and automatic fixing of errors, with (2) the Integrated Microbial Genomes Expert Review (IMG-ER) annotation pipeline (https://img.jgi.doe.gov/cgi-bin/er/main.cgi) [17]. Assigned functions were checked with BLAST [18]. Alignment and phylogenetic analysis were performed with MEGA 5.0 [19].

### Comparative genomic analysis

Artemis software was used for data management and DNAPlotter was used for genome visualization [20]. The MAUVE alignment tool was used for multiple genomes sequence alignment and visualization. IslandViewer was used to analyze possible genomic islands (GI) on the draft genome [21]. IslandViewer integrates two sequence composition GIs prediction methods, namely IslandPath-DIMOB [22] and SIGI-HMM [23] and one single comparative GI prediction method, namely IslandPick [21] for genomic island prediction.

ISsaga application from ISfinder server [24] was used to identify insertion sequences (IS) and transposons in the draft genome of LMG 26808. Sequences exhibiting homology to IS and transposon sequences were verified with the Mobilomics software [25]. The core genome was estimated using the Phylogenetic profiler tool that is part of the IMG system (https://img.jgi.doe.gov/cgi-bin/er/main.cgi) at a similarity cutoff of 50%.

The presence of possible virulence-related genes and genes expressed during tomato infection in the draft genome of the non-pathogenic *Clavibacter* was analyzed by comparing it with tomato pathogen Cmm NCPPB 382. The comparative screening of the gene content was performed in RAST, IMG-ER and EDGAR [26]. Absence or presence of coding sequences in each genome, as reported by RAST, IMG-ER and EDGAR were independently confirmed by performing protein and nucleotide BLAST queries in the target genomes. Proteins with amino acid sequence similarities higher than 50% and with a coverage higher than 70% were considered homologs. Based on the RAST, IMG-ER and EDGAR annotation results, the presence of known and putative virulence factors, pathogenicity related genes and genes uniquely present in the non-pathogenic *Clavibacter* LMG 26808 were investigated. Identification of orthologous groups between four available *Clavibacter* genomes was achieved by OrthoMCL analyses [27]. OrthoMCL clustering analyses were performed using default parameters with the P-value Cut-off = 1 × 10^−5^.

### Deposition

The current draft genome sequence was deposited at Genbank under accession number AZQZ00000000 after automatic annotation by the PGAAP online annotation pipeline.

## Results and discussion

### General features of non-pathogenic *Clavibacter* LMG 26808

Genome assembly using paired-end and mate-paired reads resulted in a 3.47 Mb sequence represented in 70 contigs from which the longest covered more than one million bp (Table 1). The initial PE *de novo* assembly was used for scaffolding with the MP dataset. In the final consensus sequence each base matched at least Phred quality score of 35. LMG 26808 contains one chromosome and evidence of a presence of a plasmid that showed a high similarity to a *Klebsiella pneumoniae* Kp11978 plasmid pOXA-48 (JN626286.1). The genes of Kp11978 were found on 15 contigs in a draft genome of LMG 26808 (estimated size of these contigs ~48 kbp,%GC ~50%) (Additional file 1: Table S1). No sequences of known *Clavibacter* plasmids could be detected. The GC content of the draft genome averages 72%. There are 46 tRNA genes and two complete rRNA operons. A total of 3218 protein-coding genes are predicted in non-pathogenic *Clavibacter* strain (in IMG-ER), which is similar to the Cmm genome NCPPB 382 that contains 3107 protein-coding genes. The genome of the non-pathogenic *Clavibacter* strain contains 685 (21.3%) proteins without predicted function being either annotated as conserved hypothetical proteins or proteins with unknown function.

**Table 1:** **Genome characteristics of the non-pathogenic** ***Clavibacter*** **LMG 26808**

Genome characteristics	Non-pathogenic ***Clavibacter*** LMG 26808
No. contigs (>200 bp)	70
Total contig size (bp)	3,476,455
N50 (bp) after scaffolding	383,456
Largest contig size (bp)	1,028,177
GC content (%)	72.01
No. RNA calls	7 rRNA
	46 tRNA
No. CDS calls	3218
NCBI accession no.	AZQZ00000000
Number of insertion elements	10

**Figure 1:**
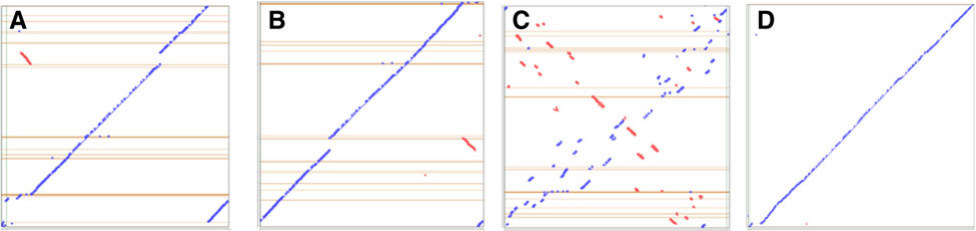
**Syntenic dotplots showing the similarity of the genomes included in the analysis. A)** non-pathogenic *Clavibacter* LMG 26808 (x-axis) and Cmm NCPPB 382 (y-axis); **B)** non-pathogenic *Clavibacter* LMG 26808 (x-axis) and Cmn NCPPB 2581 (y-axis); **C)** non-pathogenic *Clavibacter* LMG 26808 (x-axis) and Cms ATCC 33113 (y-axis); **D)** Cmm NCPPB 382 (x-axis) and Cmn NCPPB 2581 (y-axis) (Diagrams generated in IMG-ER).

The number of genes detected in the draft genome of LMG 26808 was higher than in the other three complete *Clavibacter* genomes (Table 2). The average nucleotide identity (ANI) between the draft genome of the non-pathogenic *Clavibacter* and the three published *Clavibacter* genomes Cmm NCPPB 382 (NC_009480.1), Cms ATCC 33113 (NC_010407.1) and Cmn NCPPB 2581 (NC_020891.1) was determined using the *in silico* DNA-DNA hybridization (DDH) method included in the software JSpecies [28]. The results indicated that LMG 26808 is genetically most related to Cmm NCPPB 382 (94.96% ANI), followed by Cmn NCPPB 2581 (92.75% ANI) and Cms ATCC 33113 (92.48% ANI). Although based on the ANI values the LMG 26808 genome is the most similar to that of pathogenic Cmm NCPPB 382, the synteny plots of LMG 26808 and Cmn NCPPB 2581 and the percentage of homologous genes shared by LMG 26808 and Cmn NCPPB 2581 are also considerably high (Table 2, Figure 1). The genomes of LMG 26808, NCPPB 382 and NCPPB 2581 are collinear with less than 5 recombinational breakpoints.

**Table 2:** **Comparison of genome characteristics (based on IMG-ER server)**

Genome Name	***Clavibacter michiganensis*** subps. ***michiganensis*** NCPPB 382	***Clavibacter michiganensis*** subsp. ***sepedonicus*** ATCC 33113	***Clavibacter michiganensis*** subsp***. nebraskensis*** NCPPB 2581	Non-pathogenic ***Clavibacter***LMG 26808
**Accession number** ^**a**^	NC_009480.1	NC_010407.1	NC_020891.1	AZQZ00000000
**Host**	tomato	potato	maize	*
**Disease**	bacterial wilt and canker	potato ring rot	wilt and blight	non-pathogenic
**Size**	3395237	3403786	3063596	3476455
**Genes**	3169	3168	2936	3282
**CDS**	3107	3117	2890	3218
**CDS (%)**	98.04	98.39	98.43	98.05
**RNA**	62	51	46	64
**rRNA**	6	6	6	7
**tRNA**	45	45	30	46
**Enzymes**	759	712	740	750
**CRISPR**	1	0	0	1
**GC%**	72	72	73	72
**Coding bases**	3041059	2955244	2823671	3074588
**Signalp** ^**b**^	281	234	219	140
**Signalp (%)**	8.87	7.39	7.46	4.27
**Homologs to LMG 26808 (%)** ^**c**^	2716 (87.4)	2457 (78.8)	2531 (87.5)	-

^a^ Only the Genbank records of the chromosomes are given.

^b^ Number of genes coding signal peptides.

^c^ Calculated using a Genome Gene Best Homologs tool included in IMG-ER server.

*Isolated from tomato seeds, host unknown.

Comparison on a genomic scale revealed a high conservation in the gene sequence among genomes of LMG 26808, NCPPB 382 and NCPPB 2581 (Figure 1). There are 299 genes (~10%) present in the LMG 26808 draft genome that were not detected in the Cmm NCPPB 382 genome. Forty eight of them were detected in Cmn and/or Cms genomes (Additional file 1: Table S2). 37 unique genes of LMG 26808 were associated with the plasmid and/or low GC regions. 214 of the unique genes were found in the core chromosome of LMG 26808 (Additional file 1: Table S2). Almost half of the genes specific for LMG 26808 belonged to hypothetical or unknown proteins (120). Remaining sequences were coding for additional ABC transporters, antibiotic resistance genes, acetyltransferases and several enzymes that in majority could not be assigned to any KEGG (Kyoto Encyclopedia of Genes and Genomes) pathway (Additional file 1: Table S2). When compared to other *Clavibacter* genomes, LMG 26808 appeared to not have experienced gene losses and despite it is considered only a draft, the majority of important genes involved in basic metabolism and gene regulation could be detected. Comparative analysis (based on KEGG pathways) showed that LMG 26808 lacks sulfate and nitrate reduction pathways suggesting that its capability of survival in soli might be similar to this observed in Cmm NCPPB 382. The core genome consists of 2316 homologs found in all four *Clavibacter* genomes. LMG 26808 contains 12 genomic regions exhibiting a lower GC content (Additional file 1: Table S3). Several genes coding for proteins within these regions were found to contribute to the fitness of the bacterium (Cl_02679 coding for ABC-type Fe^3+^-siderophore transport system; Cl_03044 coding for permease component, chloramphenicol acetyltransferase (EC 2.3.1.28); Cl_03094 coding for multidrug-efflux transporter). The genome heterogeneity and genetic diversity among *Clavibacter* strains most likely contribute to the differences in the bacterial lifestyle. Phage-related recombinases (e.g. Cl_00892, Cl_03056), integrase/resolvase (e.g. Cl_02713) and other insertion elements (transposases, e.g. Cl_03190) associated with a phage were found in higher numbers in the genome of LMG 26808 than in the Cmm NCPPB 382 genome (Table 3). The genome of LMG 26808 contained sixteen genes coding for transposases and recombinases (Table 3). This number was much lower in comparison to more than 100 genes found in Cms (ATCC 33113) [9]. Detected IS belonged to IS*3,* IS*4,* IS*5*, IS*6* and IS*1380* families. Transposases were represented by Tn3 (20%). No pseudogenes among transposases and recombinases were detected suggesting that these elements may encode functional genes. None of the IS elements found in LMG 26808 has homologs in the other *Clavibacter* strains. The most common IS element in Cms ATCC 33113 is IS*1121*[9]. Cmm NCPPB 382 contains only a few IS, which are most probably nonfunctional [8]. Cmn NCPPB 2581 contained only two types of IS, namely ILSre2 and ISNCY (predicted by ISsaga).

**Table 3:** **Mobile genetic elements found in the genome of LMG 26808 (Based on the annotation results from IMG-ER, RAST and EDGAR)**

CDS identifiers	COG	COG annotation	Pfam	Position	Length (bp)
Cl_00892	COG4974	Site-specific recombinase XerD, phage_integrase	pfam00589	Contig 3 (50199 to 51350)	1152
Cl_00935	COG4974	Site-specific recombinase XerD, phage_integrase	pfam00589	Contig 5 (8554 to 9540)	987
Cl_01562	COG4974	Site-specific recombinase XerD, phage_integrase	pfam00589	Contig 5 (682570 to 683556)	987
Cl_01811	COG1842	Phage shock protein A (IM30), suppresses sigma54-dependent transcription	pfam04012	Contig 5 (938633 to 939376)	744
Cl_01968	COG3600	Uncharacterized phage-associated protein	pfam13274	contig 7 (7492 to 7956)	753
Cl_03043	COG4679	Phage-related protein	pfam05973	contig 15 (974 to 1303)	330
Cl_03056	COG4974	Site-specific recombinase XerD, phage_integrase	pfam00589	contig 15 (22760 to 23485)	726
Cl_03252	COG4974	Site-specific recombinase XerD, phage_integrase	pfam00589	contig 46 (189 to 866)	678
Cl_02713	COG2452	Predicted site-specific integrase-resolvase	pfam12728	contig 11 (350076 to 350522)	447
Cl_03047	COG2801	Transposase and inactivated derivatives, Tnp1, IS3_IS150	pfam01527	contig 15 (9545 to 11077)	1533
Cl_03189	-	Transposase DDE domain, Tnp1, IS1380	pfam01609	contig 28 (1417 to 2532)	1116
Cl_03190	COG4644	Transposase and inactivated derivatives, TnpA family, Tn3	pfam01526	contig 28 (4206 to 5753)	1530
Cl_03209	COG4644	Transposase and inactivated derivatives, TnpA family, Tn3	pfam01526	contig 33 (10984 to 12801)	1818
Cl_03210	COG3316	Transposase and inactivated derivatives, IS6	pfam13610	contig 33 (12848 to 13552)	705
Cl_03211	COG2801	Transposase and inactivated derivatives, IS3_IS150	pfam13276	contig 33 (14010 to 13498)	513
Cl_03212	COG2963	Transposase and inactivated derivatives, Tnp1, IS3	pfam01527	contig 33 (14489 to 14343)	147
Cl_03214	COG3316	Transposase and inactivated derivatives, IS6	pfam13610	contig 34 (391 to 116)	276
Cl_03235	-	Transposase, Tnp1, IS5_IS903	pfam13737	contig 39 (1373 to 2212)	840
Cl_03261	-	Transposase DDE domain, Tnp1, IS4	pfam01609	contig 51 (1 to 1188)	1188
Cl_03204	-	Mobile element protein	-	contig 33 (530 to 366)	165
peg.1244	-	Mobile element protein	-	contig 28 (149 to 742)	594
Cl_03063	-	Mobile element protein	-	contig 15 (32765 to 34252)	1488
peg.807	-	Resolvase-like	-	contig 15 (6452 to 6847)	396
peg.1245	-	Tn1 transposase	-	contig 28 (741 to 1088)	348
Cl_03045	-	Gifsy-2 prophage protein	-	contig 15 (7251 to 7544)	294
Cl_03251	-	putative bacteriophage protein	-	contig 45 (3200 to 4171)	972
Cl_01918	-	elements of external origin; phage-related functions and prophages	-	contig 6 (14793 to15281)	489

The comparison of functional categories as defined by COG (Clusters of Orthologous Groups) showed noticeable differences in the gene content in categories of ‘carbohydrate transport and metabolism’ and of ‘translation, ribosomal structure and biogenesis’. All included *Clavibacter* strains contained a higher percentage of genes in these two categories than a free-living organism *Escherichia coli* 081 ED1a or a tomato pathogen as e.g. *Pseudomonas syringae* pv. *tomato* T1 (Additional file 1: Figure S1)*.* These observations are supporting the hypothesis that compared *Clavibacter* strains can most probably utilize different sugars as an energy source and that they possess a wide range of transport systems that enable the efficient trafficking of the substrates and products. The presence of a high number of genes involved in translation, ribosomal structure and biogenesis implies that these bacteria respond more effectively and rapidly to nutritional resources, which can be an important advantage in a changing environment.

### Genomic islands

The analysis of the LMG 26808 genome showed that at least 12 regions with lower GC contents distributed among different contigs could be distinguished (Additional file 1: Table S3). Parts of genomic islands 3 and 4 found in LMG 26808 partly overlap with the *chp* region of pathogenicity island (PAI) described previously in Cmm NCPPB 382 (Additional file 1: Table S3). Genomic islands with lower GC% are thought to be integrative elements that exhibit different codon usage relative to the rest of the genome, encode for transposases, integrases and are typically found at tRNA loci. Their acquisition is mostly a result of actions of phages, transposons or horizontal gene transfer [29]. Some of the genes present in these regions in LMG 26808 were detected previously in the genome of Cmm NCPPB 382 but the majority represents regions that were not found in *Clavibacter* subspecies. The total size of these regions accounts for 265 kb (~7% of the genome size). The equivalent of PAI of Cmm NCBI 382 (130 kb) containing two regions *chp* and *tomA* with important genes responsible for effective plant colonization, was not found in LMG 26808, nor in other *Clavibacter* genomes. However, a number of orthologs were found in all three *Clavibacter* genomes (Additional file 1: Table S4). The higher number of orthologs of genes encoded on chp and tomA regions (as detected by OrthoMCL) was found in Cms ATCC 33113 (32), followed by LMG 26808 (17) and Cmn NCPPB 2581 (10). Only six orthologs of PAI (*chp* region) found in LMG 26808 were located on the low GC region 3 and 4 (Additional file 1: Table S3).

Genomic regions with lower GC content can contain diverse genes exhibiting functions in many metabolic processes. The longest region found in LMG 26808 (more than 100 kb) included genes coding for antibiotic resistance (beta-lactamase class A, Cl_03208, Cl_03230), transposases (Cl_03209, Cl_03212) and many hypothetical proteins (Cl_03223, Cl_03183) some of which showed the highest similarity on the protein level to genes found on *Klebsiella pneumoniae* plasmids. Genomic region 3 contains some genes that showed similarities to the genes found in pCM2 plasmid of Cmm NCPPB 382. The majority of them are hypothetical proteins and two of them code for acetyltransferases Additional file 1: Table S3. Previous studies indicated that some pathogenic *Clavibacter* strains lacking pCM1 and pCM2 showed a positive signal in hybridization experiments with specific plasmid regions of Cmm NCPPB 382 implying that some of the genes found originally in Cmm plasmids may be actually chromosomally-encoded in other Cmm strains [30].

Genomic regions 7, 9 and 10 with lower GC content contained some genes encoding transposases and recombinases, which might imply their possible exchange/mobilization ability. In region 7 one phage-related gene (Cl_03043), showing homology to prophage protein gp49, was detected. Its presence may represent the remains of prophage genes.

### Plasmid content

LMG 26808 did not contain any of two known virulence plasmids found in pathogenic Cmm NCPPB 382. However, the plasmid extraction demonstrated the presence of one plasmid, which size was slightly smaller than that of plasmid pCM2 (70 kb). Initially, we assumed that it might be a pCM2 that lost some genes because in the previous study we could not detect the presence of the *pat-1* gene, which is encoded on the Cmm plasmid [4]. Even though we did not detect the complete pCM2, some of the genes originally encoded on this plasmid were found in LMG 26808 (Additional file 1: Table S5). Except for two genes involved in the putative conjugal transfer (pCM2_0013 and pCM_0019, coding for TraA and TraG, respectively), all the remaining genes showed to code for hypothetical or putative secreted proteins. All of them were detected on contig 6 but the order in which they were found in LMG 26808 did not match the order demonstrated in pCM2. Moreover, there are more genes present on contig 6, some of which showed to be homologous to proteins from the Cmm chromosome (Cl_01961-Cmm_02708, Cl_01957-Cmm_01374). These observations may suggest that some of these plasmid genes were incorporated in the genome of LMG 26808.

The observation that some genes from pCM2 that were expressed during tomato infection by Cmm [31] had orthologs found in LMG 26808, might suggest that although their function is unknown, they may be essential for non-pathogenic *Clavibacter* as well as pathogenic Cmm strains (Additional file 1: Table S5). Further investigation is needed to elucidate the exact functions of these genes. The smaller plasmid pCM1 was not detected during the plasmid extraction, nor were its sequences found in the genome sequence of LMG 26808. Despite that two DNA fragments of LMG 26808 showed to be highly similar to two plasmid-encoded genes, namely pCM1_0018 and pCM1_0020, the reciprocal BLAST search revealed that these sequences from the non-pathogenic *Clavibacter* genome are more similar to the chromosomally encoded CMM_1065 and CMM_2443, respectively. Interestingly, the latter gene encodes CelB, which is a putative secreted cellulase that contains a cellulose-binding domain (endo-1,4-beta-glucanase). Chromosomally encoded c*elB* misses one of three protein coding domains present in the *celA* gene. The missing endoglucanase C-terminal domain is similar to the α-expansin protein family that occurs in plants and is essential for development of wilting and for degradation of crystalline cellulose [8, 32]. The lack or disruption of any of these domains of *celA* inevitably led to the disability to induce disease symptoms in a tomato plant [32].

Surprisingly, the genome analysis showed the presence of sequences found in *Klebsiella pneumoniae* plasmid pOXA-48 (61881 bp). The presence of sequences from a plasmid of Gram-negative bacteria in a Gram-positive *Clavibacter* strain is rather unusual and has not been reported previously. Although the genome sequence of LMG 26808 is only a draft and therefore incomplete, we could not detect any sequences that could be attributed to a *Klebsiella pneumoniae* Kp11978 chromosome.

The exchange of genetic material between various prokaryotes is well known and has been extensively studied over the last few decades [33–37]. Although it was demonstrated for bacteria that the gene exchange is observed more frequently between closely related genera with a similar GC content and exhibiting high sequence similarities there are examples of recent gene transfers between distantly related bacteria (e.g. Actinobacteria and gammaproteobacteria) [38].

Conjugational transfer is considered the most efficient way of LGT [39, 40] that contribute the most to the spread of antibiotic resistance among different bacteria [41]. This type of LGT is widely encountered among various bacterial species and even between bacteria and Archaea [42] on the one hand and between bacteria and higher organisms such as *Saccharomyces cerevisiae*[43], or plants [44] on the other hand. Conjugational plasmid exchange was also observed within the genus *Clavibacter* in which the endophytic CMM100 strain (cured of pCM1 and pCM2 plasmids) was able to acquire these plasmids from other pathogenic Cmm strains and restore pathogenicity [45]. Furthermore, transformation experiments carried out with *Clavibacter xyli* subsp*. cynodontis* (currently reclassified to the genus *Leifsonia*) demonstrated the possibility to acquire an IncP plasmid from Enterobacteriaceae by this Gram-positive Actinobacteria, which provided another evidence of conjugational transfer between diverse taxa [46].

*Klebsiella pneumoniae* strains were found in many important crops such as potato, maize, soybean, cotton and tomato [47, 48]. Many of these strains carry plasmids that contain antibiotic resistance genes and possess the conjugation transfer systems which enable the gene mobilization and exchange among and outside Enterobacteriaceae and other bacterial genera [49]. Some genes encoded on the *Klebsiella pneumoniae* plasmids exhibit high similarities to regions found previously in *Escherichia coli* and *Yersinia* genomes, implying that there is an active genetic exchange among strains of these genera [50].

Although an acquisition by LMG 26808 of a relatively large plasmid originating most probably from a member of *Enterobacteriaceae*/*Klebsiella* relative by LMG 26808 (Additional file 1: Figure S2) was unexpected and unprecedented, a similar occurrence was previously reported by Baltrus and coworkers. They detected a recent acquisition of a megaplasmid by two cucumber isolates of *Pseudomonas lachrymans*[51]. It was suggested that this acquisition resulted from an important ecological shift across closely related *Pseudomonas* members and that the plasmid-encoded genes may be advantageous for the recipient bacteria.

As *Klebsiella pneumoniae* and *Clavibacter* strains thrive in the same environmental niche (associated with tomato) and because of examples of possible genetic material exchange between distantly related bacteria we can hypothesize that the acquisition of plasmid sequences encoding antibiotic resistance genes might provide a competitive advantage for the non-pathogenic *Clavibacter* strain LMG 26808.

### Non-pathogenic lifestyle

Non-pathogenic *Clavibacter* strains from tomato seeds tested in the previous study [4] as well as other strains tested by Jacques and coworkers [3] did not introduce any disease symptoms when tested on tomato plants. Possible explanations for the non-pathogenic nature of these strains are i) the lack of two plasmids present in pathogenic Cmm and carrying virulence factors, ii) the absence of the pathogenicity island and iii) a significantly lower number of genes coding for extracellular hydrolytic enzymes including several important serine proteases, glycosyl hydrolases and the plant cell wall-hydrolyzing enzymes.

In pathogenic Cmm*,* main virulence factors *cel-A* and *pat-1*, encoded on pCM1 and pCM2, respectively, are required to induce disease symptoms (wilting and canker) in tomato plants [52, 32]. Moreover, genes coding for the production of extracellular enzymes, such as endoglucanase, polygalacturonase, xylanase, serine proteases and other secreted proteins have been implicated as possible virulence factors in recent reports [30, 31, 52, 53]. The genome of LMG 26808 did not contain the most prominent virulence factors *pat-1* and *celA*. Their absence may be directly linked with the absence of the pCM1 and pCM2 plasmids in the non-pathogenic *Clavibacter*. However, southern hybridization experiments with plasmid fragments containing virulence factors showed that in some plasmid-free pathogenic Cmm strains these virulence determinants had homologues on the chromosome [30].

A proteomic study of tomato-Cmm interaction identified several bacterial proteins with a putative role in signal perception, transduction and response to impulses. They belong to two-component system proteins, transcriptional regulators and other DNA binding proteins. They are believed to play a role in sensing the tomato plant environment and initiating pathways, possibly leading to disease development [31]. All putative genes encoding proteins that are probably involved in signal exchange between tomato and bacterium could be identified in the genome sequence of LMG 26808 (Additional file 1: Table S6).

As a non-pathogenic bacterium, LMG 26808 was hypothesized to contain less genomic information for hydrolytic enzymes that are known to be expressed during tomato infection with Cmm [31]. As expected, the most important group containing genes coding for secreted proteases from Pat-1 family was largely absent in LMG 26808 (Additional file 1: Table S6). Additional *pat-1* homologues encoded on the pCM2 plasmid (plasmid homologs of *pat-1*, *phpA* and *phpB*) were also absent. From seven genes encoding putative serine proteases *chpA-chpG* (chromosomal homologs of *pat-1*) [54] only sequences similar to *chpF* and *chpG* were detected (*chpF* and *chpG* are orthologs with nucleic acid sequence similarity of 69.1% and amino acids sequence similarity of 68%). Both these sequences, however, matched to the same region and a reciprocal best BLAST hit confirmed the presence of only *chpF.* Interestingly, the lack of *chpG* may be a possible explanation for the disability of LMG 26808 to produce a HR in nonhost plants since the *chpG* mutant in Cmm was unable to cause an HR in *Mirabilis jalapa*[8]. Moreover, the low colonization efficiency of LMG 26808 could be attributed to the lack of the *chpC* gene. A *chpC* mutation in the pathogenic Cmm NCPPB 382 resulted in a drastic reduction in colonization abilities in tomato plants [8, 55]. Pseudogenes *chpA, chpB* and *chpD* were not found in LMG 26808. The family of *chp* genes is important for plant-pathogen interaction in Cmm, but probably also in Cms where four orthologs were found. Cmn genome had no orthologs of these genes.

The majority of members of secreted serine proteases of the Ppa family (PpaA-PpaJ) that are encoded in several different loci on the chromosome and on pCM1 could not be found in LMG 26808. Orthologs of *ppaI* and *ppaF* were found in LMG 26808. Cms ATCC 33113 contained orthologs of *ppaB1, ppaB2, ppaF*, *ppaI, ppaA* in the chromosome and *ppaC* on pCS1 plasmid. On the contrary Cmn NCPPB 2581 had only one ortholog of *ppaF*. Since *ppaA* and *ppaC* genes are important for plant colonization [8] and they were absent in LMG 26808, it can be another evidence why non-pathogenic *Clavibacter* strains are poorly colonizing tomato plants. Indeed, secreted serine proteases studied in pathogenic Cmm are thought to presumably facilitate the interaction between Cmm and its host plant and are believed to play a function in pathogenicity by a possible utilization of plant proteins [31]. Their lack might imply that interaction between LMG 26808 and tomato is actually very limited. *TomA* gene of Cmm NCPPB 382 (CMM_0090), encoding endo-1,4- beta galactosidase involved in detoxification of the alfa-tomatine, had orthologs in three other *Clavibacter* genomes. However, the similarity based on the amino acid sequence was rather low (coverage (%)/ identity (%): 47/24 in Cms, 47/23 in Cmn and 47/22 in LMG 26808).

Genes coding for subtilases *sbtA, sbtB* and *sbtC* are known to be secreted during the plant infection [31]. Orthologs of these three subtilases genes were found in all four *Clavibacter* genomes. Sbt proteins of Cmm are highly similar to different tomato subtilases, some of which have been associated with wound formation and pathogen responses [56] and may play a role in the disease development. Because they are present in the non-pathogenic *Clavibacter* strain their function probably cannot be solely associated with the disease development. Cellulases and pectinases are the most important enzymes degrading plant cell walls. In many bacteria genes encoding these enzymes were found to be virulence determinants [57]. In the genome of LMG 26808 genes for pectate lyases, *pelA1* and *pelA2* and cellulase *celA* were not found. However, another cellulase *celB*, the polygalacturonase *pgaA* (whose substrate is pectin), *xysA* (whose substrate is β-1,4-xylan) and an arylesterase (which hydrolyzes ester bonds between hemicelluloses and lignin) [58] were present in LMG 26808 (Additional file 1: Table S6). These findings support the thesis that the non-pathogenic *Clavibacter* strain is probably less efficient in digesting pectins and cellulose into simpler by-products than the pathogenic Cmm that is equipped with many various plant cell degrading enzymes.

Enzymes from a large group of glycosyl hydrolases (GH) which hydrolyze the glycosidic bond between two carbohydrates or between a carbohydrate and a noncarbohydrate molecule [59] were also expressed during plant infection of Cmm. Therefore, many of them are assumed to be potential virulence factors that can hydrolyze substrates of plant origin [31]. Our results demonstrated that genes for the majority of these enzymes are present in LMG 26808 suggesting that their function might not be restricted to disease development alone. The glycosyl hydrolases are not considered as bona fide virulence factors, but as reflecting the adaptation to the differing composition of nutrients in planta allowing the survival inside of the plant.

Very important functions involved in transport and metabolism are linked to the presence of ABC and other transporters that ensure the uptake of amino acids, metals, sugars, oligopeptides, etc. Some of these transporters that were expressed during tomato infection by Cmm may utilize plant molecules to support its metabolism. The genes found in the genome of LMG 26808 that code for transporters that are known to be expressed during plant infection by Cmm are listed in Additional file 1: Table S7. Interestingly, only five orthologs of fifty seven transporters could not be found in LMG 26808. Furthermore, the genome of non-pathogenic *Clavibacter* contained additional transporters that were not present in the pathogenic Cmm genome (Additional file 1: Table S2). Some of them are supposed to play a role in the active drug transport and cell protection from toxic metabolites (C_03094 and Cl_03219). Another very important example of additional ABC transporters in the genome of LMG 26808 (not found in other three *Clavibacter* genomes) are transporters involved in iron complex transport (ABC-type Fe^3+^ siderophore transport system Cl_02679 and ABC-type cobalamin/Fe3 + −siderophores Cl_ 02677) (Additional file 1: Table S8). An alternative iron uptake system found in LMG 26808 might be advantageous in an iron deficient environment. This data suggests that LMG 26808 is probably able to utilize a broad variety of compounds to maximize its survival changes. Many environmental bacteria were shown to contain a high number of transporter genes in support of an environmental lifestyle [60].

Observations described above correlate well with the initial assumptions that suggested that non-pathogenic *Clavibacter* strains must have lost or never contained prominent virulence determinants responsible for disease induction in tomato plants. These hypotheses were partially underpinned by similar findings in another draft genome of non-pathogenic *Clavibacter* LMG 26811, which lacks the majority of virulence factors including two main determinants. It also contained less plant cell degrading enzymes than pathogenic Cmm NCPPB 382 (data not shown). Furthermore, the comparative genome analysis of LMG 26808 and Cmm NCPPB 382 revealed that some putative virulence factors, determined based on expression levels obtained from the proteomic study of tomato-Cmm interaction [31], were also present in LMG 26808, which may indicate their redundant functions and suggest that they are not critical for Cmm virulence.

### Survival in the environmental niche

Non-pathogenic *Clavibacter* strain LMG 26808 was isolated from tomato seeds yet knowledge on its environmental niche is largely lacking. Ecological niche(s) and transmission routes have not yet been found. Preliminary results with colonization experiments showed poor colonization of vascular tissues of tomato and seemingly lower survival potential of LMG 26808 in comparison to Cmm [4]. The HR was not induced in *Mirabilis jalapa* (J. Van Vaerenbergh, data not published), indicating that non-pathogenic *Clavibacter* strains do not contain genes that would be recognized by the plant to trigger the active plant defense response.

*Antibiotic resistance*. In the genome of LMG 26808 several additional genes responsible for antibiotic resistance were detected (Additional file 1: Table S2). They coded for beta-lactamases (Cl_03263, peg.1233, peg.1766, peg.1776), chloramphenicol acetyltransferase (Cl_03044) and tetracycline efflux protein TetA (peg.1764). They showed the highest similarity to genes found in *Klebsiella pneumonia, Escherichia coli* and *Salmonella enterica* suggesting that they could originate from these bacteria. In addition to the above genes, the genome of LMG 26808 contains two drug efflux transporters (Cl_03219, Cl_03094) not found in pathogenic Cmm NCPPB 382. Interestingly, LMG 26808 contained glyoxalase/bleomycin resistance protein (Cl_03100), which probably constitutes the resistance to bleomycin-antibiotic produced by some *Streptomyces* strains [61].

The presence of additional acetyltransferases might suggest that LMG 26808 exhibits broad resistance to certain antibiotics as some of the acetyltransferases (GNAT superfamily) catalyze the selective acetylation of one of the four amino groups found on a diverse set of aminoglycosides with antibiotic properties [62]. Acetylation reduces the affinity of these compounds for the acceptor tRNA site on the 30S ribosome. As a consequence, bacteria expressing these genes are resistant to some aminoglycosides with antibiotic properties. The ability to cope with antibiotics produced by organisms with which non-pathogenic *Clavibacter* strains share the environmental niche is a significant adaptive advantage. The growth of *Clavibacter* strains in culture is often inhibited by other faster growing organisms. Therefore, the presence of genes coding for antibiotic resistance might be the reason why non-pathogenic *Clavibacter* strains are more frequently encountered and isolated from the semi-specific medium during the tomato seed certification.

*Toxin-antitoxin system.* The presence of the toxin-antitoxin (T-A) genes (YefM Cl_00198, peg.1235 and YoeB Cl_00197) in the genome of non-pathogenic *Clavibacter* is intriguing and raises questions concerning their origin and potential functions in relation to the physiology of the bacterium (Additional file 1: Table S2). The YefM and YoeB T-A genes were found in many bacterial genomes and sometimes more than one copy per genome [63]. It was demonstrated that T-A systems are present only in environmental and free-living organisms and were not detected in intracellular bacteria [63]. The BLASTp analysis of YefM and YoeB genes from LMG 26808 revealed high similarities to proteins from *Rhodococcus pyridinivorans* AK37 and *Microbacterium testaceum* StLB037, respectively*.* The T-A system found in LMG 26808 was not present in the pathogenic Cmm, but YefM (peg.1235) was present in another non-pathogenic *Clavibacter* strain LMG 26811 (data not shown). Interestingly, Cmn contained another putative toxin-antitoxin system. T-A systems are not essential for normal cell growth, nevertheless they are present in many bacteria and Archaea [63]. Based on the frequency of T-A systems, it was suggested that they play subtle roles that are advantageous for cell survival in their natural habitats. Toxins may facilitate cellular adaptation of an organism to changing environments by slowing down its cell growth, inhibiting its cell growth, or causing some of its cells to die [64]. It is possible that the presence of a T-A system in the LMG 26808 genome increases the fitness of this bacterium in the occupied environmental niche. Differences in the detected toxin-antitoxin systems in particular *Clavibacter* subspecies might be attributed to different ecological niches and inhabited hosts.

*Error prone UmuDC operon*. SOS mutagenesis response in bacteria includes error-prone and error-free DNA damage repair responses that are activated after exposure to different antibiotics, chemical compounds or radiation [65]. In *Escherichia coli UmuDC* proteins are involved in error-prone bypass of UV lesions and UmuC proteins possess DNA polymerase activity. In the SOS process many genes get induced and their products are involved in DNA repair, replication and cell cycle control in order to repair the DNA damage [66]. The genes coding for this operon were found in LMG 26808 and also in another non-pathogenic *Clavibacter*, LMG 26811 (data not shown), implying that their cells might have higher abilities to recover after exposures to UV and/or other types of chemicals retrieved during the seed certification procedures. Some of the sequences coding for genes of *UmuC* operon (e.g. peg.1211) and antibiotic resistance genes (e.g. Cl_03263) described above are associated with the plasmid and/or low GC regions (Additional file 1: Table S2).

*The extracellular polysaccharide (EPS).* The genomes of all four analyzed *Clavibacter* strains contained four gene clusters involved in exopolysaccharides production (Additional file 1: Table S9). The EPS production in LMG 26808 is expected to effectively occur since all genes involved in that process are functional (no frameshifts, no pseudogenes). LMG 26808 contains almost a complete set of genes involved in the EPS production described in Cmm NCPPB 382. There is, however, one notable difference between pathogenic Cmm NCPPB 382 and LMG 26808. In the EPS2 of LMG 26808 the order of the genes is disrupted because they are located at different contigs. The functionality of this gene cluster is therefore unknown. Even if functional, it will probably be dependent on different regulation factors which may eventually influence the EPS production. EPS gene clusters in the pathogenic Cms underwent quite some drastic changes with disruptions by insertion elements and most likely the EPS production in Cms ATCC 33113 is limited. EPS clusters 1, 3 and 4 are also complete in Cmn NCPPB 2581. In case of the EPS cluster 2 in Cmn NCPPB 2581 there are three additional genes (CMN_00784, CMN_00787, and CMN_00792) located in between other genes (Additional file 1: Table S9). The main EPS composition of Cmm and Cms strains was determined experimentally and it showed some differences even though the general structure of a repeating unit of four sugars seems to be the same [67]. The number of hydrolases in Cms ATCC 33113 and Cmm NCPPB 382 is the same but some of them show lower similarities indicating differing substrate specificity/a different sugar incorporated. The composition of EPS in LMG 26808 was not yet experimentally determined. Even though this non-pathogenic strain showed a very similar genetic structure of EPS clusters to those of Cmm NCPPB 382 some genes have lower similarities (e.g.: polysaccharide polymerase (Wzy2-70% similarity; Wzy1-73% similarity); glucosyl transferases (WcmL-75% similarity; WcqR-70% similarity)). These findings indicate that the composition of EPS might be different between Cmm and non-pathogenic strains. In many bacteria the ability to produce EPS and their presence in the cell wall surface has been shown to participate in the interaction between bacteria and the environment. EPS is believed to prevent bacterial attachment to host cells which in consequence prevents the recognition of the bacteria by the host and the induction of an HR reaction [68]. In many plant pathogens EPS production prevents bacterial immobilization by host lectins and in that way allows bacteria to spread in the xylem vessels [67]. The presence of at least three functional EPS gene clusters in LMG 26808 might facilitate the EPS production in different environmental conditions and can be an advantage for non-pathogenic *Clavibacter,* which probably inhabits not only tomato seeds but also other environmental niches.

## Conclusions

The analysis of the genome sequence of the non-pathogenic *Clavibacter* LMG 26808 revealed that this strain is adapted to a non-pathogenic lifestyle. This is reflected by the lack of prominent virulence factors present in pathogenic Cmm and by the presence of a significantly lower number of genes encoding enzymes involved in digesting plant material and extracellular proteins that are potential virulence determinants. Also, LMG 26808 contained many transport proteins and transcriptional regulators implying its capacity to utilize various compounds and to respond rapidly to a changing environment. The genome of LMG 26808 contained also a high number of ABC transporters and genes involved in the cell signalling (comparable to those from some free-living bacteria).

The draft genome of the non-pathogenic *Clavibacter* strain and the comparative analysis with other whole *Clavibacter* genomes provided valuable insights into the genetic bases of pathogenicity and mechanisms involved in the adaptation to host plants and to environmental niches. Our results demonstrated that some of the putative virulence factors were also present in LMG 26808, which suggests that these genes rather contribute to the general fitness (iron uptake systems, proteases) of the bacterium by increasing competitiveness and adaptive abilities in the same environment than playing a role in virulence. Whether the non-pathogenic *Clavibacter* strain can turn into a pathogen will depend not only on the presence of additional fitness genes that allow for efficient host colonization and adaptation, but mainly on the presence of functional virulence genes. LMG 26808 does not contain *celA* and *pat-1*, the two most important virulence factors and lacks some other important determinants contributing to the effective plant colonization and involved in cell maceration and degradation. This specific combination of features likely represents the basis of its nature as a free-living bacterium and might exhibit the possible evolutionary process that involves horizontal gene transfer and gene loss, which shaped this bacterium into a non-pathogen. Because the diversity of non-pathogenic *Clavibacter* strains investigated so far is much higher than observed for Cmm it will be very interesting to investigate more of these strains in order to reveal the common genetic features and to determine factors responsible for their non-pathogenic nature. So far, some of the genomic adaptations, such as the presence of additional antibiotic resistance genes and a toxin-antitoxin system could be confirmed in a draft genome sequence of another non-pathogenic *Clavibacter* strain (data unpublished). A more in-depth comparative analysis with newly sequenced *Clavibacter* genomes will allow generating more knowledge about underlying biology of these bacteria and enabling the selection of group-specific regions that will serve as targets for development of reliable identification primers for novel control strategies.

The availability of genome sequences of *Clavibacter* strains is a critical to understanding of the processes involved in the evolution of these subspecies and in gaining more insight into the genetic basis of their pathogenic and non-pathogenic nature. Our findings confirmed the thesis that the non-pathogenic *Clavibacter* strain contains specific fitness factors but lacks crucial virulence determinants, which likely contribute to its poor colonization abilities and survival in the tomato plant. The comparison of Cmm and a non-pathogenic *Clavibacter* strains demonstrated that it is difficult to define real virulence factors since some of the genes previously assigned as putative virulence factors for Cmm are also present in the non-pathogenic strain. The role of many putative virulence factors is not clear, which partially can be attributed to the functional redundancy of these genes and to the complex and not well understood processes of their regulation. In consequence, it is dependent on the environmental niche and growth conditions (pathogen inside the host versus non-pathogenic strain in the environment) whether the presence of additional factors increasing the general strain fitness will contribute to virulence.

## Electronic supplementary material

Additional file 1: Table S1: Genes of plasmid pOXA-48 from a *Klebsiella pneumoniae* (Kp) strain Kp11978 (JN626286.1) found in the genome of LMG 26808 (Based on the BLASTn and BLASTp results). **Table S2**: List of 299 genes found in the LMG 26808 genome but not present in Cmm NCPPB 382. **Table S3**: Low GC regions of LMG 26808 detected by IslandViewer and their orthologs in other *Clavibacter* genomes. **Table S4**: Orthologs of genes encoded on chp and tomA regions of PAI of Cmm 382 found in other *Clavibacter* genomes (based on OrthoMCL). **Table S5**: List of genes from Cmm plasmids pCM1 and pCM2 found in the genome of non-pathogenic *Clavibacter* strain LMG 26808. **Table S6**: A list of Cmm genes containing known and putative virulence factors and other bacterial genes that are possibly involved in functions such as signal perception and transduction and interaction with tomato plant (as described in the publication of Savidor et al. [31]) and their orthologs in other *Clavibacter* genomes. **Table S7**: The list of transporters expressed in planta during the tomato infection by Cmm (Savidor et al, [31]) and their homologs and orthologs in LMG 26808. **Table S8**: Genes of LMG 26808 involved in iron acquisition and metabolism (based on COG groups from IMG-ER) and their orthologs in other *Clavibacter* genomes. **Table S9**: The extracellular polysaccharide (EPS) gene clusters present in *Clavibacter* genomes. **Figure S1**: Percentage of the total number of genes in each functional category as defined by COG (clusters of orthologous groups). The analysis was performed in IMG-ER. **Figure S2**: Plasmid extraction of LMG 26808 (arrow points at the plasmid of LMG 26808). (XLSX 165 KB)
